# Prevalence of maternal burn out and depression in tunisia during the coronavirus pandemic

**DOI:** 10.1192/j.eurpsy.2021.701

**Published:** 2021-08-13

**Authors:** E. Bergaoui, R. Lansari, A. Karmous, A. Larnaout, W. Melki

**Affiliations:** Psychiatry D, Razi Hospital, Manouba, Tunisia

**Keywords:** coronavirus, lockdown, maternal burn out, Depression

## Abstract

**Introduction:**

Lockdown due to coronavirus pandemic has been a stressful experience especially to mothers. Juggling work from home and childcare has led to maternal burn out and depression.

**Objectives:**

The aim of the present study was to assess maternal burn-out rate during lockdown and its eventual relationship with depression and associated factors

**Methods:**

156 Tunisian mothers responded to online questionnaire posted on social network after 1 month of lockdown. The questionnaire evaluated burnout and depression as measured by the Maslach Burnout Inventory (MBI) and depression and anxiety symptoms (HADS) respectively.

**Results:**

The participants were aged between 24 and 64 years and 61.5% had more than one child. Mean score on the BMS10 was 4.11 out of 7 maximum score with 71.8% of participants fulfilling criteria for maternal burn-out. Among them, 30.1% had high level of burn out and 9.6% extreme burn out. The main factors associated with maternal burn out were age of children, financial difficulties and lack of leisure activities. Mother’s age, perceived husband support, medical or psychiatric history haven’t been associated with maternal burn out. HADS questionnaire indicated that 38.5% of mothers had moderate to severe anxiety disorder and 35,9% had moderate to severe depressive disorder. A postive correlation was found between burnout and anxiety (r=0.634, p<0.001) and burn out and depression (r=0.515, p<0.001).

**Conclusions:**

The prevalence of maternal burn out during lockdown was significantly high resulting in higher rate of depression than ususal. However, severe forms of burnout may share several characteristics with depression raising the question of overlap of these two entities.

